# High risk Langerhans cell histiocytosis in children: the role of salvage in improving the outcome. A single center experience

**DOI:** 10.1186/s13023-024-03232-8

**Published:** 2024-06-24

**Authors:** Mohamed Sedky M. Sedky, Asmaa Hamoda, Hala Taha, Iman Zaky, Omayma Hassanain, Alaa ElHaddad

**Affiliations:** Children’s Cancer Hospital Egypt 57357 and National Cancer Institute, 1, Sekket Al-Imam St., Al Sayyeda Zeinab, Cairo, 26386 Egypt

**Keywords:** 2-CdA based regimen, High risk organs, LCH, Disease progression, Reactivation

## Abstract

**Background:**

In pediatric multi-system high risk organs (RO +) Langerhans cell histiocytosis (LCH), failing 1st line treatment has the highest mortality. We aim to present the outcome of failure of 1st line whether due to disease progression (DP) at end of induction or reactivation (REA) after initial better status response.

**Patients and methods:**

Sixty-seven RO + LCH patients with hemopoietic, hepatic or splenic involvement, treated between 2007 and 2019 were retrospectively analyzed. The median follow-up (IQR) is 6 years (4–8.8 y).They were subjected to 2 eras of treatment; one with salvage by 2-Cda based regimen (2-CdABR) and another without.

**Results:**

Of 67 patients, M/F 40/27, median age 1.74 y (0.2–10 y), 42 failed 1st line (62.7%). Of them DP *n* = 22 (52%) and REA *n* = 20 (48%). Of those with DP, 9/22 patients received 2-CdABR, where 5 survived in better status. While the remaining 13 did not receive 2-CdABR and all of them died. Otherwise, of those with REA, 12/20 reactivated on RO + mode. Of them, 8/12 received 2-CdABR, where only one survived in better status and the remaining 4 received vinblastine-based regimen,where 2 died and 2 were rescued. RO + 5-year overall survival (OS) was 65% (CI 95% 54 -78) while the event free survival (EFS) 36% (26.3—50.1). The OS of DP 27% (14–54) versus REA 67% (49–93) p 0.004. OS of DP with 2-CdABR 56% (31–97.7) versus 8% without (2–51), *p* < 0.001. While OS of REA with 2-CdABR 38% (13–100) versus 74% without (53–100) p 0.7.

**Conclusion:**

Survival of RO + remains limited. Failure of 1st line in RO + due to DP carries worse prognosis in relation to REA. In DP those who were not salvaged by 2-CdABR, showed dismal outcome. This could not be shown when applied in REA.

**Supplementary Information:**

The online version contains supplementary material available at 10.1186/s13023-024-03232-8.

## Introduction

Langerhans cell histiocytosis (LCH) is a myeloid neoplasm with a high inflammatory part [1, 2]. The worst outcome is associated with the involvement of high-risk organ (RO + LCH) including the hematopoietic system, liver and spleen [3]. The addition of intermediate dose methotrexate (ID MTX) did not improve the results [4]. For this, it has been omitted in the more recent LCH IV protocol [5]. Subsequently, failure of 1st line treatment either by DP or REA remains accountable for a dismal outcome with the least survival reaching 30% [6–8]. For this, the LCH IV protocol has offered the purine analogue 2-chlordeoxyadenosine 2- CdA as a 2nd line salvage treatment [5]. This drug has undergone a potential modification of its indication after the breakthrough innovative anti B RAF and anti MEK targeted therapy [9–13].

Selection of anti-cancer therapy in low middle income countries has become a priority in oncology management [14]. Hereby we present the outcome of salvage of RO + LCH in a pediatric Egyptian single center population treated over a period of more than 12 years.

## Patients and methods

It is a single center retrospective analysis of pediatric LCH patients aged from birth to 18 years old treated at Children Cancer Hospital—Egypt 57,357. Diagnosis and stratification were confirmed according to the Histiocyte Society (HS) [5, 15–17]. 

### Study population 

The medical records of 74 LCH multisystem RO + organ (MSRO +) patients treated at Children cancer hospital Egypt CCHE 57357 between July 2007 and end December 2019 were retrospectively reviewed and analyzed after the approval of the scientific and medical advisory (SMAC) and the institutional review board (IRB) committees. They were treated in the RO + group of the histiocyte society protocols with LCH III protocol [4] starting from 2007 till mid- 2012 and LCH IV from 2012 to end 2019 [5]. Of them, 7 patients were excluded either as they died during the 1st 2 weeks of diagnosis, or they were lacking complete data. We retrieved 67 RO + patients that were subjected to history and physical examination of the initial disease, the response to first line treatment, various prognostic factors, and the outcome of salvage 2nd line treatment.

### Stratification 

High-risk organs patients were stratified when any of the Multisystem “RISK” organs (RO +) involvement was confirmed according to Lahey criteria: hematopoietic system with cytopenias (Bi, Tri/cytopenia) defined as anemia (hemoglobin: < 100 g/L, infants < 90 g/L, and or leucopenia, white blood cell count < 4 × 10E9/L, and or thrombocytopenia platelets < 100 × 10E9/L or BMA infiltrated by positive CD1a [16, 17]. Hepatomegaly was described as a size of at least 3 cm below the costal margin and/or hepatic dysfunction (hypoproteinemia, hypoalbuminemia, hyperbilirubinemia, and/or increased liver enzymes). Splenomegaly was described as at least 2 cm below the costal margin. Both hepatomegaly and splenomegaly were confirmed by ultrasound. Lung involvement was confirmed with the presence of cysts or nodules on computed tomography radiologic examination [18, 19]. Evaluation at the end of induction post week 12 was decisive for the response [4, 5].

### Treatment failure

Either disease progression (DP) or reactivation (REA) was an indicator for a treatment failure. DP was recorded, if the patient showed worse status {progressive active disease worse (ADW) or stationary active disease intermediate (ADI)} to induction treatment. REA was recorded if the patient showed worse status after having achieved a better status by the end of the induction phase [4, 20, 21]. Organ Failure to 1st line was present whether by DP or REA of cytopenia, hepatic involvement (hepatomegaly and or hepatic dysfunction), or splenomegaly.

### Salvage

While adopting the LCH III protocol, treatment failure necessitated a 2nd line treatment [4]. In DP no second line could be offered, and the patient was treated on palliative or compassionate basis. Otherwise in REA, management was by a repetition of 1st line induction PRED / VBL ± ID MTX molecules. Adopting the LCH IV [5] -to DP or REA on a RO + mode- offered a 2nd line salvage including 2-CdA based regimen (2-CdABR) [5, 22].

### Fate of salvage

RO + The response was assessed according to organ hemopoietic or hepatic or splenic involvement and was divided into either better status (NAD or ADB) or worse status (ADI or ADW) by last follow up.

### Toxicity to salvage

2-CdABR was assessed according to OMS toxicity scoring system to induce morbidity and related mortality [23].

### Prognostic factors

The following factors to affect survival (OS or EFS) were assessed: (1) age category below or above 2 years; (2) gender male versus female; (3) hemopoietic cytopenia versus none (4) hepatic involvement versus none; (5) splenomegaly versus none (6) 1st line ID MTX including regimen versus none (7) DP versus REA, (8) REA low-risk versus high-risk mode, 9) 2-CdABR vs no 2-CdABR in failure 1st line, (10) 2-CdABR vs none in DP. 11) 2-CdABR vs none in REA.

### Data collection and statistical analysis

Quantitative variables were summarized using the median and range, while qualitative variables using crude frequencies. Five-year Overall survival (OS) starts from date of diagnosis till time of death, or last follow up. Event-free survival (EFS) is defined as time from diagnosis till disease progression (DP), reactivation REA, death, or last follow up. While to compare salvage treatments, (OS) was defined as time from failure to initial 1st line treatment either DP or REA until death, or last follow up. Survival analysis was conducted using Kaplan–Meier function, and curves compared using the log-rank test. Competing risk analysis was done using the cumulative incidence function (CIF) and Gray’s test. All analyses were conducted using R version 4.1.0. *P*-values ≤ 0.05 were indicative of statistical significance and, tendency to be statistically significant if between 0.05 and 0.1.

## Results

### Clinical characteristics

Of the retrieved 67 RO + patients, M/F ratio was 47/20 and the median age 1.74 y (0.2–10.1 y). They were analyzed for the outcome of treatment from 8/2007 to 4/2019. The median follow-up (IQR) is 6 years (4 to 8.8). Their demography is shown in Table 1.

**Table 1:** Patients characteristics and impact on 5-year OS and EFS

	**Number**	**%**	**OS**	**CI**	***P* value**	**EFS**	**CI**	***P* value**
**Gender**								
Male	40	60%	60%	47–78	0.3	51%	38–70	0.4
Female	27	40%	71%	56–90		66%	50–87	
**Median age**	1.74 (0.2–10.1)							
**Age group**								
< 2 y	44	66%	57.8%	44.6—74.8	0.2	51%	38–69	0.03
> 2 y	23	34%	78.3%	63.1—97.1		69%	52–91	
**Cytopenia**	20	30%	44%	26.2—72.6	0.02	31.0	15—62	0.05
**No cytopenia**	47	70%	73%	60.8—87.2		66.5	54 -82	
**Hepatic**	51	76%	56%	43–72	0.02	48%	36–64	0.03
**No hepatic**	16	24%	92%	77–100		83%	65–100	
**Splenomegaly**	45	67%	57%	44–74	0.1	47%	34–65	0.06
**No Splenomegaly**	18	33%	82%	77–100		77%	66–97	
**1st line**								
**LCH III** **VBL/ PREDs/IDMTX**	20	30%	75%	58–97	0.5	75%	58–97	0.01
**LCH IV** **VBL/ PRED**	47	70%	61%	48–77		49%	36–66	

*CI* Confidence interval, *EFS* Event free survival, *ID MTX* Intermediate dose methotrexate, *OS* Overall survival, *PRED* Prednisone, *VBL* Vinblastine

### Clinical stratification

Hematopoietic involvement with cytopenia was present in 20 patients and hepatic involvement with hepatomegaly and or hepatic dysfunction in 51 patients, while splenomegaly was present in 45 patients.

### 1st line treatment

Twenty patients received LCH III vinblastine/ prednisone /intermediate dose methotrexate (VSM) (30%). Otherwise, 47 received LCH IV vinblastine/ prednisone (VS) (70%). The clinical characteristics of the patients are shown in Table 1.

### Failure of 1st line treatment

**Fig. 1:**
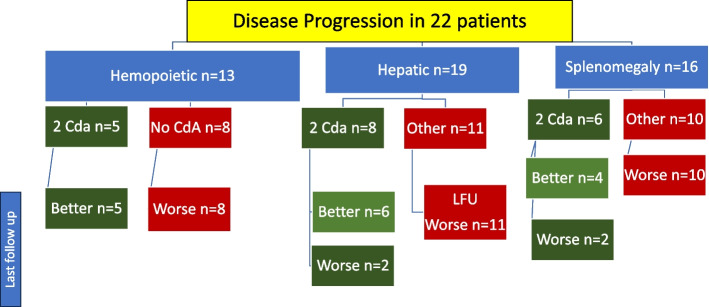
**a** Outcome of disease progression per organ in RO+ involvement according to treatment by 2-Cda based regimen. **b** Outcome of reactivation per organ in RO+ involvement according to treatment by 2-Cda based regimen

**Fig. 2:**
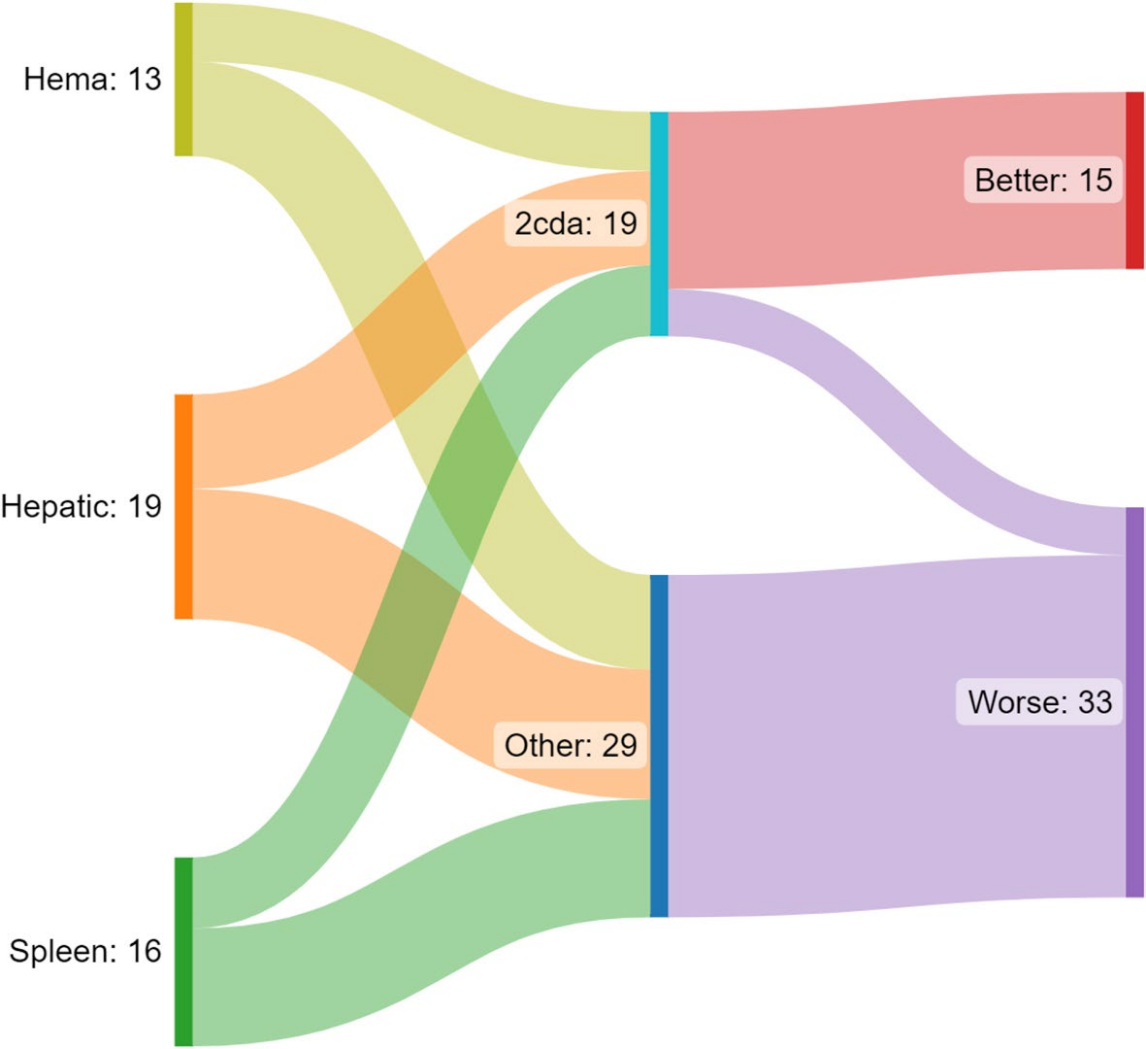
**a** Fate of 2-CdA and other salvage in Disease Progression per RO+ hematopoietic (Hema), hepatic and spleen. At last follow up, all 19 RO+ involvement who received 2 CdA showed better status except 4 . All who did not receive 2 CdA showed worse status at last follow up. **b** Fate of 2 CdA and other salvage in reactivation per RO+ hematopoietic (Hema), hepatic and splenic At last follopw up, All 19 RO+ involvement who received 2 CdA showed worse status except 2. All 9 RO+ who received other salvage showed worse status except 2

Assessment of first line treatment showed failure in 42 patients (63%) where DP *n* = 22 (52%): 13 hemopoietic (59%) 19 hepatic (86%) and 16 splenic (73%). While REA *n* = 20 (48%):7 hemopoietic (35%), 9 hepatic (45%). and 12 splenic (60%) Fig. 1 (a, b) and 2 (a, b).

**Fig. 3:**
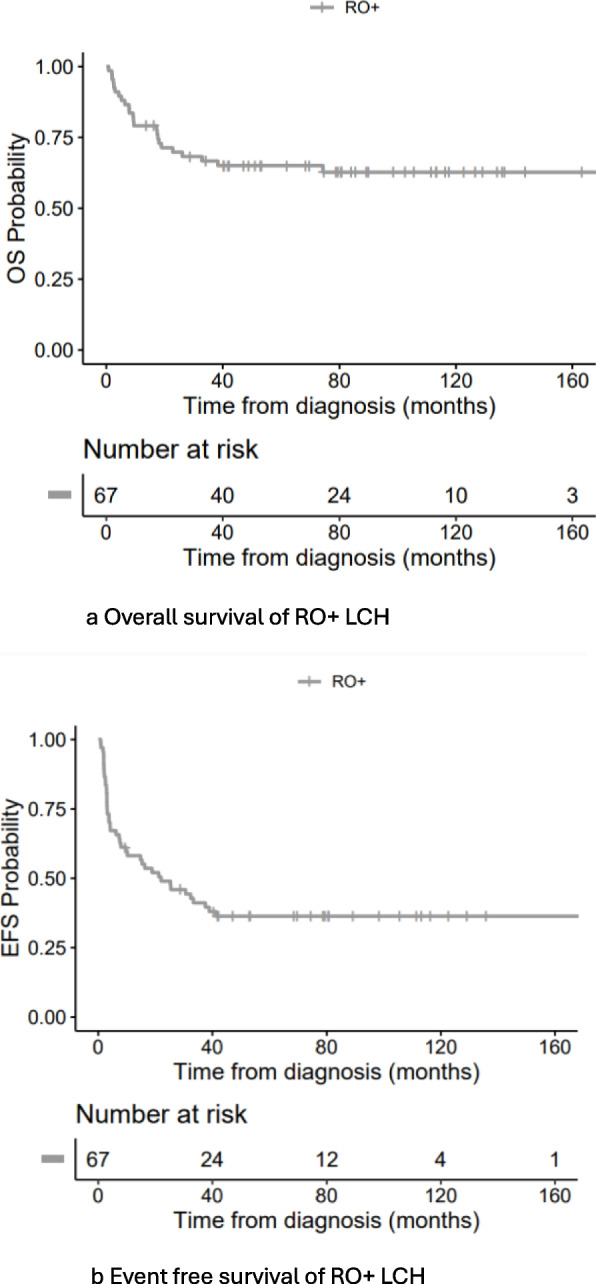
** a** Overall survival of RO+ LCH. **b** Event free survival of RO+ LCH

The 5-year cumulative incidence % of DP was 35.7% (95% CI 21.7—49.9) in VS versus 15.0% (3.6 -34.0) in VSM: Gray’s test *P*-value = 0.07. While that of REA was 15.5% (6.7—27.6) in VS versus VSM 10.0% (1.6—27.9) by Gray’s test P3-value = 0.44. Salvage for disease progression (SDP): Of the 22 patients showing DP to 1st line induction, salvage could not induce better status at last follow up except in 5 out of the 9 patients who received 2-CdABR of the LCH IV protocol. All other patients who received either compassionate basis or palliative treatment, showed worse status at last follow up. As regard RO + response, this ‘Better Status’ at last follow up was achieved in: hemopoietic *n* = 5/5, Hepatic *n* = 6/8 splenic *n* = 4/6. The details of fate of 2-CdABR per RO + involvement is shown in Figs. 1 (a) and 2 (a) and Supplement table S1a. Salvage for Reactivation (SREA): Of the 20 patients showing REA, RO- was the site of reactivation in 8 patients (40%). They received salvage by repeated 1st line treatment including vinblastine prednisolone with or without methotrexate and all survived except one reactivating on histiocytic sarcoma (HS) (UPN 3) Supplement table S1b. Otherwise, 12 patients (60%) showed RO + REA whether hemopoietic, hepatic or splenic. All of them received 2nd line salvage treatment with or without 2-CdABR. This 2-CdABR did not lead to a noticeable better status at last follow up. Out of those 8 patients, only one patient survived in better status at last follow up, 3 patients were alive in worse status and 4 patients died. As regard RO + response, this ‘Better Status’ at last follow up was achieved in hematopoietic *n* = 1/5, Hepatic *n* = 0/6, splenic *n* = 1/8. The details of fate of 2-CdABR per RO + involvement is shown in Figs. 1 (b) and 2 (b) and Supplement table S1b. 2-CdA related mortality: Seventeen patients received 2-CdABR. Out of 9 patients in DP, 3 patients showed pneumonia, where 2 died (UPNPRO 13 and 15) in ‘Better Status’. Otherwise, out of 8 patients in REA, one patient showed pancreatitis and proctitis (UPNREA 20) Supplement table S1. Survival: For 67 RO + , the 5-year OS was 65% (CI 95% 54 -78), while the EFS was 36% (26.3—50.1) Fig. 3 (a, b). We had a lower OS survival with hemopoietic cytopenia and hepatic involvement. Similarly, the EFS was lower by age less than 2 years, hematopoietic cytopenia, hepatic involvement and 1st line regimen non including ID MTX. All these results are statistically significant and are shown in Table 1.

**Fig. 4:**
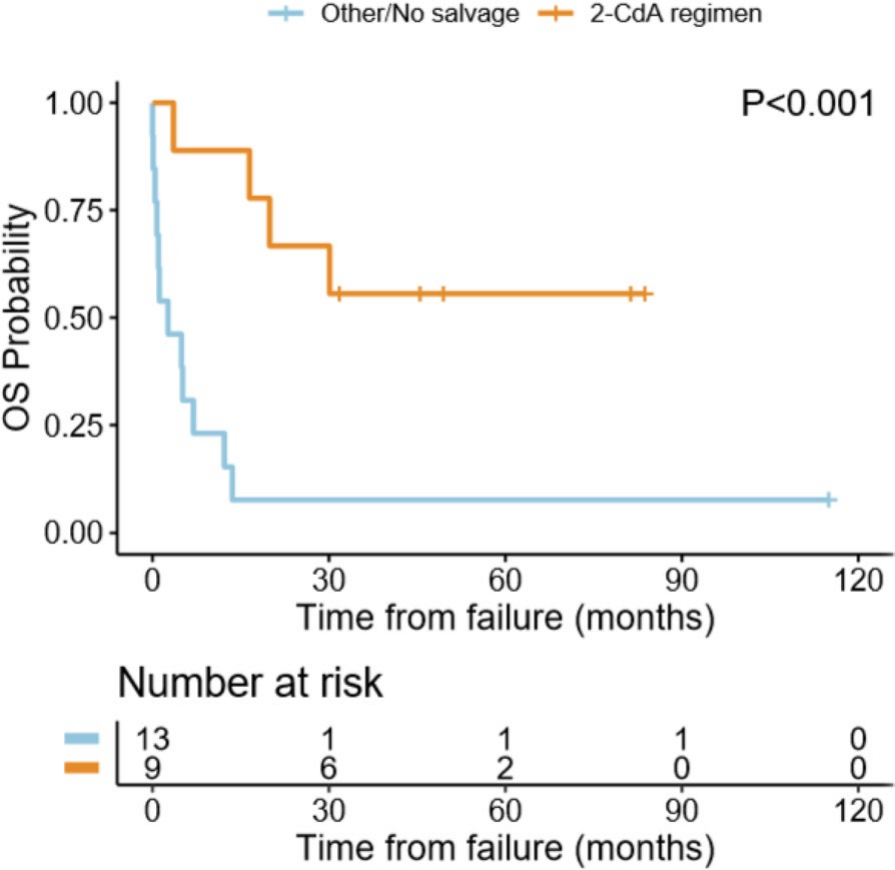
Overall survival of disease progression 2-CdA salvage

The 5- year OS in DP was 27%, versus 67% in REA p 0004 with a lesser OS 46% if reactivation on RO + mode in relation to OS 100% if reactivation on RO- mode p 0.008 Table 2. When assessing the impact of 2-CdABR salvage on survival in 42 patients showing failure to treatment, the OS in 22 DP patients per 2-CdABR vs none was 56% and 8% respectively *p* < 0.001 Table 2, Fig. 4. On the other hand, the OS in 20 REA patients per 2-CdABR vs none was 38% versus 74% respectively p 0.07 Table 2.

**Table 2:** 1st line treatment failure and salvage impact on 5 year overall survival

	Number	%	OS	CI	*P*
DP	22	30%	27%	14–54	0.004
REA	20	33%	67%	49–93	
Risk organ REA					
RO-	7	35%	100%	NA	0.008
RO +	13	60%	46%	24–87	
2-CDA in failure	17	40%	45%	24–81	0.4
No 2-CdA in failure	25	60%	40%	24–65	
2-CDA in DP	9	41%	56%	31–97.7	< 0.001
No 2-CdA in DP	13	59%	8%	2–51	
2-CDA in REA	8	40%	38%	13–100	0.07
No 2-CdA in REA	12	60%	74%	53–100	

*REA* Reactivation, *DP* Disease progression, *RO-* Low risk organs, *RO* + High risk organs, *2-CdA* 2chloredeoxyadenosine

## Discussion

RO + pediatric LCH, carries a bad prognosis in patients failing the 1st line treatment [3]. Patients were divided into two groups according to the period and the treatment they received. Those through LCH III protocol, who received treatment before mid-2012 and having ID MTX during induction but without salvage by 2-Cda. Otherwise, those through LCH IV protocol who received treatment after 2012 without ID MTX in induction but with 2-CdABR salvage treatment; a regimen that has been proved to be effective, but toxic [22]. Nowadays as RO + LCH frequently harbor the *BRAF*^*V600E*^ mutation [24], the innovative anti B RAF targeted therapy is becoming in common use in refractory RO + [13, 25]. However, being an off-label, expensive drug with a higher reactivation risk at discontinuation, anti B Raf is becoming a useful option as a 3^ed^ line treatment [25].

ID MTX in LCH III protocol did not prove any effectiveness on survival on the long term, reason for which it has been omitted afterwards in the subsequent LCH IV protocol [4]. In our study, there was a rise in the cumulative incidence of DP in patients not receiving ID MTX with a tendency to be statistically significant. The present updated analysis with 67 patients is more prominent than our previous one which comprised a smaller number of 50 patients [21]. Since pediatric LCH is a rare disease, it is difficult to statistically empower such a comparison at a reasonable alpha error rate. On the other hand, ID MTX was associated with better EFS with statistically significant results, confirming our previous study and others [21, 26].

Failing induction DP RO + comprise most early deaths [6, 22, 27]. Otherwise, disease REA, although rare in RO + , is still responsible of mortality in a small number of patients [28]. In our study, DP was accompanied by the worst OS in relation to REA *p* < 0.001. This is even worse if REA occurred in RO + organs p 0.008, reflecting the benignity of RO- responding to repetition of 1st line treatment. While 2-CdABR was provided starting from 2012, OS in DP with 2-CdABR was 56% vs 8% without p 0.004. This statistically proves the high regimen efficiency with the best response in patients with hematopoietic system involvement. All these patients showed better status with 2-CdABR at last follow up, differently from hepato-splenic DP that responded partially to 2-CdABR. This could be partially explained by the presence of a resistant sclerosing cholangitis. Donadieu et al. showed the prompt effect of 2 CdA aracytine in a refractory RO + cohort -excluding sclerosing cholangitis- in a phase II LCH-S-2005 study [22]. It showed an overall response rate (ORR: NAD and ADB) of 92% and a 5-year OS 85% contrarily to a previous study LCH-S-98 study revealing the lack of disease control by 2-CdA monotherapy with a 2-year OS of 48.0% [10]. In an updated nationwide survey from 2005 to 2019 in Japan, Tanigushi et al. showed 21 RO + patients failing 1st line, where the ORR was 50% the OS of 86% and EFS of 77% [12].

In our series, the role of 2-CdABR could not be proven in REA with even a worse survival than other salvage lines; this probably due to considerable REA on RO- mode responding to repeating 1st line treatment. In REA on RO + mode, neither 2-CdABR or other chemotherapy salvage regimen could control the disease promptly. In this situation 2-CdABR is even too toxic in this hepatic sclerosing cholangitis form; a more severe resistant disease presenting at a later stage reaching biliary cirrhosis and hepatic cell failure beyond the scope of chemotherapy. Otherwise, cytopenia and splenomegaly as a RO + mode of reactivation, responded exceptionally to both 2-CdABR and other salvages; but these results remain subjective. Outcome of 2- CdA on REA remains variable as Imamura showed a limited outcome if REA occurred on RO + mode [11] contrarily to the possibility of rescuing RO + twice, the first on REA RO + liver and spleen and the other on REA RO- [29].

In our series, 2-CdA based regimen was responsible of severe infectious complications mainly in those with DP, showing pneumonia in 1/3 of them. Moreover, 2 patients out of 3 died from infection in a better status as regard the initial disease. Otherwise infection was exceptional in REA -in the form of proctitis and pancreatitis- and was of favorable outcome. As 2-CdA causes lymphopenia, and prolonged pancytopenia [30], Donadieu et al. showed that 2- CdA induced immune suppression caused half of the deaths from fulminant viral infection [22].

The incidence of cancer deaths is 70% in low/middle-income countries (LMICs) [31]. This is partly due to the lack of chemotherapeutic agents [14]. In this optic, we investigated the outcome of RO + for proper treatment planning according to cost effectiveness. In our early practice, between 2007 and 2012 we could not use 2- CdA, an expensive drug lacking the evidence, and this led to the mortality of all RO + failing 1st line LCHIII protocol. Between 2012 and end 2019, 2-CdABR rescued all DP RO + patients with hematopoietic involvement and most of hepatosplenic ones. JSLG 02 protocol offers a salvage treatment including cyclophosphamide, doxorubicin, Vincristine, prednisone cyclosporin A in RO + patients failing 1st line treatment. However, its results are non-satisfactory with survival reaching 70% requiring the use of further 2-CdA or targeted therapy [32]. In low-income countries, further salvage treatment beyond first-line therapy for LCH remains economically difficult. Therefore, to improve survival, reducing 1st line treatment failure remains strategic by augmenting induction and prolonging maintenance in RO + patients. Narula et al., showed that RO + LCH receiving oral etoposide augmented induction and maintenance had early and durable responses. Prolonging maintenance including methotrexate lowered reactivation rates in RO + and RO − LCH, resulting in excellent survival and reduced the need for salvage [33]. In our population, it is still hard to conclude the superiority of ID MTX with confidence due to the unbalanced 2 subgroups of a relatively small population. This would invite the re discussion of the role of ID MTX in decreasing DP at end of induction. If ascertained, this would reduce the need for 2- CdA as a salvage line and thus offer favorable cost-effective management for a potentially lethal pediatric RO + LCH.

## In conclusion

Survival of RO + LCH remains limited. Failure of 1st line in RO + due to DP carries worse prognosis in relation to REA. Those who were not salvaged by 2-CdABR in DP showed dismal outcome. This could not be shown when applied in REA. There is a certain role of ID MTX in reducing cumulative incidence of DP of induction and thus better EFS. Our recommendations are to restrict 2-CdA to DP rather than REA especially those with late stage hepatic involvement with criteria of sclerosing cholangitis.

### Supplementary Information

Supplementary Material 1.

## Data Availability

Through electronic medical records “Cerner” and the datasets used/analyzed during this study are available from the corresponding author on request. The data that support the findings of this study are not openly available due to reasons of sensitivity and are available from the corresponding author upon reasonable request.

## Abbreviations

ADB: Active disease better
ADI: Active disease intermediate
ADW: Active disease worse
ARAC: Aracytine
BMA: Bone marrow aspirate
BRAF: B-Raf murine sarcoma viral oncogene homolog B
2-CdA: 2-chlordeoxyadenosine
2-CdABR: 2-Chlordeoxyadenosine based regimen
CIF: Cumulative incidence function
DP: Disease progression
EFS: Event-free survival
HS: Histiocyte Society
ID MTX: Intermediate dose methotrexate
IRB: Institutional review board
LCH: Langerhans cell histiocytosis
LMIC: Low/middle-income countries
M/F ratio: Male: female ratio
MSRO +: Multisystem risk Organ
NAD: No active disease
OS: Overall survival
PRED: Prednisone
REA: Reactivation
RO +: High risk organ
SDP: Salvage for disease progression
SMAC: Scientific medical advisory committee
SREA: Salvage for reactivation
VBL: Vinblastine
VMF: Vemurafenib
