# Supplementary material for: High risk Langerhans cell histiocytosis in children: the role of salvage in improving the outcome. A single center experience
Source: Orphanet J Rare Dis. 2024 Jun 24;19:242. doi: 10.1186/s13023-024-03232-8 (PMC11195005; doi:10.1186/s13023-024-03232-8)
Supplement: Supplementary file 1 — Supplementary Material 1. [file 13023_2024_3232_MOESM1_ESM.pdf]

Supplementary Table S1a: Fate of Disease progression to 1st line treatment.

| UPN | Pro CTPN | Pro hep dysf | Pro hmg | Pro smg | Type therpro | Respindsalv | CumDos | 2cdaRM | Fail2cda | Organ | Treat Failure | Fate CTPN | Fate hepdys | Fate hmg | Fate smg | LFU | COD |
|---|---|---|---|---|---|---|---|---|---|---|---|---|---|---|---|---|---|
| 1 | + | + | - | - | No salvage | No salvage | No2-CdA | No2-CdA | No2-CdA | No2-CdA | No2-CdA | Worse | Worse | Nopro | Nopro | DIED | DP |
| 2 | - | + | + | + | No salvage | No salvage | No2-CdA | No2-CdA | No2-CdA | No2-CdA | No2-CdA | nopro | Worse | Worse | Worse | DIED | DP |
| 3 | + | + | + | + | HLH | ADW | No2-CdA | No2-CdA | No2-CdA | No2-CdA | No2-CdA | Worse | Worse | Worse | Worse | DIED | DP |
| 4 | - | - | + | - | COMPR.PNT | ADB | No2-CdA | No2-CdA | No2-CdA | No2-CdA | No2-CdA | nopro | nopro | Better | Nopro | ALIVE | NO |
| 5 | + | - | + | + | COMPR. | ADW | No2-CdA | No2-CdA | No2-CdA | No2-CdA | No2-CdA | Worse | Worse | Stationary | Worse | DIED | DP |
| 6 | + | + | + | + | HLH | ADW | No2-CdA | No2-CdA | No2-CdA | No2-CdA | No2-CdA | Worse | Worse | Worse | Worse | DIED | DP |
| 7 | + | + | - | + | FLU/ARAC | ADW | No2-CdA | No2-CdA | No2-CdA | No2-CdA | No2-CdA | Worse | Worse | Worse | Worse | DIED | DP |
| 8 | + | + | + | +/- | No salvage | No salvage | No2-CdA | No2-CdA | No2-CdA | No2-CdA | No2-CdA | Worse | Worse | Worse | Worse | DIED | DP |
| 9 | - | + | - | - | 2cda/ARAC | ADB | 150 mg/m2 | NO | REARO+ | SC | Palliative | Worse | Worse | Worse | Worse | DIED | Liver failure |
| 10 | - | + | - | - | No salvage | No salvage | No2-CdA | No2-CdA | No2-CdA | No2-CdA | No2-CdA | Worse | Worse | Worse | Worse | DIED | DP |
| 11 | + | + | + | - | 2cda/ARAC | ADB | 120 mg/m2 | NO | NO | NO | NO | Better | Better | Better | Nopro | ALIVE | NO |
| 12 | - | + | - | - | 2cda/ARAC | ADB | 120 mg/m2 | NO | NO | NO | NO | Nopro | Better | Nopro | Nopro | ALIVE | NO |
| 13 | + | + | + | + | 2cda/ARAC | ADB | 120 mg/m2 | viral pneumonia | REARO- | Bone, Skin | VBL/Ste | Better | Better | Better | Better | DIED | Septic shock |
| 14 | - | + | + | + | 2cda/ARAC | ADW | 45 mg/m2 | NO | PRORO+ | SC | Palliative | nopro | Worse | Worse | Worse | DIED | liver failure |
| 15 | + | + | - | - | 2cda/ARAC | ADB | 90 mg/m2 | pneumonia | NO | NO | NO | Better | Better | Nopro | Nopro | DIED | Pneumonia |
| 16 | + | +/- | +/- | +/- | No salvage | ADW | No2-CdA | No2-CdA | No2-CdA | No2-CdA | No2-CdA | Worse | Worse | Worse | Worse | DIED | DP |
| 17 | - | + | + | - | Palliative | ADW | No2-CdA | No2-CdA | No2-CdA | No2-CdA | No2-CdA | Worse | Worse | Worse | Nopro | DIED | DP |
| 18 | + | - | + | + | 2cda/ARAC | NAD | 120 mg/m2 | pneumonia | NO | NO | NO | NAD | nopro | NAD | NAD | ALIVE | NO |
| 19 | + | + | - | +/- | No salvage | No salvage | No2-CdA | No2-CdA | No2-CdA | No2-CdA | No2-CdA | Worse | Worse | Nopro | Worse | DIED | DP |
| 20 | + | + | - | - | 2cda/ARAC | ADB | 120 mg/m2 | NO | REARO- | Bone, Skin | VCR/ARAC/Ste | Better | Better | Nopro | Nopro | ALIVE | NO |
| 21 | - | + | + | + | Palliative | ADW | No2-CdA | No2-CdA | No2-CdA | No2-CdA | No2-CdA | Nopro | Worse | Worse | Worse | DIED | DP |
| 22 | - | + | + | - | 2cda/ARAC | ADB | 120 mg/m2 | NO | REARO- | SC | VBL/Ste | Nopro | Better | Better | worse | ALIVE | For liver transplant |

ADB: active disease better, ADW active disease worse, 2cdaRM: 2-CdA related mortality, COD cause of death, COMPR compressed cycle, CumDos; cumulative doses, fail 2cda: failure of 2cda, Fate CTPN: fate cytopenia, Fate hepdysf: fate hepatic dysfunction, Fate hmg fate hepatomegaly, Fate smg fate splenomegaly, FLU/ARAC fludarabine aracytine, HLH: hemophagocytosis lymphohistiocytosis, LFU: last follow-up status, NAD: no active disease, PNT purinethol, Pro CTPN: progressive cytopenia, Pro hep dysf: progressive hepatic dysfunction, Pro hmg: progressive hepatomegaly, Pro smg: progressive splenomegaly, Respindsalv; response to salvage induction, SC sclerosing cholangitis, type therpro: type therapy to disease, treatfail; treatment failure, UPN: unique patient number, VBL vinblastine.

Supplementary Table S1b: Fate of reactivation of 1st line treatment.

| UPN | numrea | RISK | Orgrea | REA CTPN | REA hepdysf | REA HMG | REA SMG | Salvrea | resp | CumDos | 2cdaRM | Fail2cda | Fate CTPN | Fate hepdysf | Fate hmg | Fate smg | LFU | COD |
|---|---|---|---|---|---|---|---|---|---|---|---|---|---|---|---|---|---|---|
| 1 | 1 | RO- | Post Pituit, Skin | - | - | - | - | VBL/PRED/MTX | ADB | no2cda | No2cda | no2cda | NoREA | NoREA | NoREA | NoREA | Alive | NO |
| 2 | 1 | RO- | Orbit | - | - | - | - | VBL/PRED/MTX | ADB | no2cda | No2cda | no2cda | NoREA | NoREA | NoREA | NoREA | Alive | NO |
| 3 | 1 | HS | LN/Bone | - | - | - | - | AML protocol | ADW | no2cda | No2cda | no2cda | NoREA | NoREA | NoREA | NoREA | DIED | DP |
| 4 | 1 | RO- | Bones, Lung | - | - | - | - | VBL/PRED/MTX | ADB | no2cda | No2cda | no2cda | NoREA | NoREA | NoREA | NoREA | Alive | NO |
| 5 | 1 | RO+ | Cytopenias, Hepatosplenic | + | + | + | + | VBL/PRED/MTX +HLH | ADW | no2cda | No2cda | no2cda | Worse | Worse | Worse | Worse | DIED | DP |
| 6 | 2 | RO- | Bones, orbit | - | - | - | - | VBL/PRED/MTX | ADB | no2cda | No2cda | no2cda | NoREA | NoREA | NoREA | NoREA | Alive | NO |
| 7 | 1 | RO+ | HSM, Bones, LN,Lung | - | - | + | + | VBL/PRED/MTX | ADB | no2cda | No2cda | no2cda | NoREA | NoREA | Better | Better | Alive | NO |
| 8 | 2 | RO- | Bones | - | - | - | - | VBL/PRED x2 | ADB | no2cda | No2cda | no2cda | NoREA | NoREA | NoREA | NoREA | Alive | NO |
| 9 | 4 | RO+ | Hepatosplenic, Bone, Skin | - | + | + | + | VBL/PRED x2 | ADB | no2cda | No2cda | no2cda | NoREA | Better | Better | Better | Alive | NO |
| 10 | 2 | RO+ | Cytopenia, hepatosplenic | + | + | + | + | VBL/PRED/MTX +HLH | ADW | no2cda | No2cda | no2cda | Worse | Worse | Worse | Worse | DIED | DP |
| 11 | 1 | RO- | Bones | - | - | - | - | VBL/PRED/MTX | ADB | no2cda | No2cda | no2cda | NoREA | NoREA | NoREA | NoREA | Alive | NO |
| 12 | 1 | RO+ | Cytopenia, Splenomegaly | + | - | - | + | 2cda/ARAC | ADW | 45 mg/m2 | CTPN/ PNM/ sepsis/ICU | PRORO+ | Worse | NoREA | NoREA | Worse | DIED | DP |
| 13 | 1 | RO+ | BM, bone hepatosplenic | + | + | + | + | 2cda/ARAC | ADW | 150 mg/m2 | Sepsis/ICU H1N1 PNM | PRORO+ | Worse | Worse | Worse | Worse | DIED | DP |
| 14 | 1 | RO- | LN | - | - | - | - | VBL/PRED | ADB | no2cda | No2cda | no2cda | NoREA | NoREA | NoREA | NoREA | Alive | NO |
| 15 | 1 | RO+ | Hepatosplenic, LN, Bones | - | + | + | + | 2cda/ARAC | ADW | 45 mg/m2 | PNM ARDS ICU | PRORO+ | Worse | Worse | Worse | Worse | DIED | DP |
| 16 | 1 | RO+ | cytopenia, lung hepatosplenic, LN | + | - | + | + | 2cda/ARAC | ADB | 120 mg/m2 | No | NO | Better | NoREA | Better | Better | Alive | NO |
| 17 | 1 | RO+ | Liver/DI/Skin | - | + | + | + | 2cda/ARAC | ADB | 120 mg/m2 | No | NO | Worse | Worse | Worse | Worse | ALIVE & worse | NO |
| 18 | 1 | RO+ | Hepatosplenic, LN, Bones | - | + | + | + | 2cda/ARAC | ADW | 120 mg/m2 | NO | PRORO+ | NoREA | Worse | Worse | Worse | ALIVE & worse | NO |
| 19 | 1 | RO+ | HMP/ hepatosplenic | + | + | + | + | 2cda/ARAC | ADW | 90 mg/m2 | Sepsis, ICU Brady | PRORO+ | Worse | Worse | Worse | Worse | DIED | DP |
| 20 | 1 | RO+ | HMP/ hepatosplenic | + | + | + | Yes | 2cda/ARAC | ADW | 90 mg/m2 | Pancreatitis, Proctitis, | DC | Worse | Worse | Worse | Worse | ALIVE & worse | NO |

ADB: active disease better, ADW active disease worse, ARAC aracytine, ARDS acute respiratory distress syndrome, HLH: hemophagocytosis lymphohistiocytosis, numrea: number of reactivations, Organ organ reactivation, REA CTPN: reactivation cytopenia, REA Hep dysf: reactivation hepatic dysfunction, REA hmg: reactivation hepatomegaly, REA smg: reactivation splenomegaly, salvREA salvage to reactivation, CumDos; cumulative doses, Fail2cda, failure 2cda, 2cda related mortality, Fate CTPN: fate cytopenia, Fate hepdysf fate hepatic dysfunction, Fate hmg fate hepatomegaly, Fate smg fate splenomegaly, LFU last follow-up status, PNM pneumonia, ICU intensive care unit, COD cause of death, UPN: unique patient number, VBL vinblastine.
